# Antibacterial, Anticandidal, and Antibiofilm Potential of Fenchone: In Vitro, Molecular Docking and In Silico/ADMET Study

**DOI:** 10.3390/plants11182395

**Published:** 2022-09-14

**Authors:** Wasim Ahmad, Mohammad Azam Ansari, Mohammad Yusuf, Mohd Amir, Shadma Wahab, Prawez Alam, Mohammad N. Alomary, Abdulrahman A. Alhuwayri, Maria Khan, Abuzer Ali, Musarrat Husain Warsi, Kamran Ashraf, Maksood Ali

**Affiliations:** 1 Department of Pharmacy, Mohammed Al-Mana College for Medical Sciences, Dammam 34222, Saudi Arabia; 2 Department of Epidemic Disease Research, Institute for Research and Medical Consultations (IRMC), Imam Abdulrahman Bin Faisal University, Dammam 31441, Saudi Arabia; 3 Department of Clinical Pharmacy, College of Pharmacy, Taif University, Taif 21944, Saudi Arabia; 4 Department of Natural Products and Alternative Medicine, College of Clinical Pharmacy, Imam Abdul Rahman bin Faisal University, Dammam 31441, Saudi Arabia; 5 Department of Pharmacognosy, College of Pharmacy, King Khalid University, Abha 61421, Saudi Arabia; 6 Department of Pharmacognosy, College of Pharmacy, Prince-Sattam Bin-Abdulaziz University, Al-Kharj 11942, Saudi Arabia; 7 National Centre for Biotechnology, King Abdulaziz City for Science and Technology (KACST), Riyadh 11442, Saudi Arabia; 8 Maternity and Children Hospital, Buraydah 51452, Saudi Arabia; 9 Department of Pharmacognosy, R.V. Northland Institute, Dadri 203207, India; 10 Department of Pharmacognosy, College of Pharmacy, Taif University, Taif 21944, Saudi Arabia; 11 Department of Pharmaceutics and Industrial Pharmacy, College of Pharmacy, Taif University, Taif 21944, Saudi Arabia; 12 Faculty of Pharmacy, Universiti Teknologi MARA (UiTM), Cawangan Selangor, Kampus Puncak Alam, Bandar Puncak Alam 42300, Selangor Darul Ehsan, Malaysia; 13 Department of Pharmacognosy, Orlean College of Pharmacy 42, Knowledge Park—III, Greater Noida 201308, India

**Keywords:** fenchone, biofilm, essential oil, antimicrobial activity, molecular docking

## Abstract

The aim of the present study is to investigate the effective antimicrobial and antibiofilm properties of fenchone, a biologically active bicyclic monoterpene, against infections caused by bacteria and *Candida* spp. The interactions between fenchone and three distinct proteins from *Escherichia coli* (β-ketoacyl acyl carrier protein synthase), *Candida albicans* (1, 3-β–D-glucan synthase), and *Pseudomonas aeruginosa* (Anthranilate-CoA ligase) were predicted using molecular docking and in silico/ADMET methods. Further, to validate the in-silico prediction, the antibacterial and antifungal potential of fenchone was evaluated against *E. coli*, *P. aeruginosa,* and *C. albicans* by determining minimum inhibitory concentration (MIC), minimum bacterial concentration (MBC), and minimum fungicidal concentration (MFC). The lowest MIC/MBC values of fenchone against *E. coli* and *P. aeruginosa* obtained was 8.3 ± 3.6/25 ± 0.0 and 266.6 ± 115.4/533.3 ± 230.9 mg/mL, respectively, whereas the MIC/MFC value for *C. albicans* was found to be 41.6 ± 14.4/83.3 ± 28.8 mg/mL. It was observed that fenchone has a significant effect on antimicrobial activity (*p* < 0.05). Our findings demonstrated that fenchone at 1 mg/mL significantly reduced the production of biofilm (*p* < 0.001) in *E. coli*, *P. aeruginosa,* and *C. albicans* by 70.03, 64.72, and 61.71%, respectively, in a dose-dependent manner when compared to control. Based on these results, it has been suggested that the essential oil from plants can be a great source of pharmaceutical ingredients for developing new antimicrobial drugs.

## 1. Introduction

Antibiotics are the essential medication for a variety of microbial infections with the goal of improving life expectancy [1]. Several antibiotic groups, such as cephalosporin, carbapenems, and aminoglycosides, which had better efficacy, have become less effective against certain infections due to increased drug-resistant nature in organisms. There is an urge to investigate newer drugs with less resistance. Fenchone, a bicyclic monoterpene ketone [2], is the most abundant compound present in *Foeniculum vulgare* oil [3], which is important for larvicidal and fungicidal properties in the seed [4]. Fenchone also has anti-inflammatory [5], anti-hyperglycemia [6], antioxidant [7], and antinociceptive properties [8]. The major causative agent for morbidity and mortality worldwide is *Candida* species [9], which causes vulvovaginitis, oropharyngeal, skin candidiasis, candidemia (infection in the blood), and infectious diseases [10]. The common aggressive pathogen which causes invasive fungal infections in hospitalized individuals is *Candida albicans* [11]. The cellular membrane of *C. albicans* has a two-layered morphology with a β-glucan-chitin framework because β-1,3-glucans are the most widely used molecules [12]. Most antifungal drugs show a toxic effect on both fungi and the host which leads to adverse effects such as anorexia, dizziness, cirrhosis, dermatitis, skin eruption, constipation, vomiting, headaches, and digestive problems [13]. Targeting the cell wall components which are not present in mammals is considered to be effective in treating fungal infections [14,15]. The integrity of the cellular membrane is maintained by enzyme 1, 3-β–D-glucan synthase, which is important for fungal cell division and proliferation [16] and about 65–85% of the glucan is present in 1, 3-β–D glucan [17].

*P. aeruginosa* is a particularly antibiotic-resistant Gram-negative common bacterium, due to reduced membrane permeability, biochemical drug deactivation through gene transfer, and biofilm formation [18,19]. Around 65% of patient mortality and antibiotic resistance is mainly due to *P. aeruginosa* [20]. Additionally, *P. aeruginosa’s* ability to form biofilm also hinders infection therapy by protecting them from environmental stressors, impeding phagocytosis, and conferring colonization and long-term persistence [21]. Such ability is promoted by quorum sensing, a cell-to-cell communication mechanism that plays an important role in the formation of highly structured biofilm in *P. aeruginosa* [22,23]. Biofilms are responsible for over 90% of persistent wound infections, causing poor wound healing [23]. Quorum sensing plays a vital role in establishing persistent infections [24], which is often focused on the processing, secretion, and recognition of tiny soluble quorum-detecting transcription factors [25]. Because the Pseudomonas quinolone signal is stronger [26], and PqsA is necessary for its production [27], inhibiting the enzymes may disrupt biosynthesis. The anthranilate-CoA ligase enzymes mostly expressed by the pqsA gene can be utilized as a target to predict a potent molecule to impede biofilm formation by *P. aeruginosa* [28].

Only a few strains of *E. coli*, a Gram-negative bacterium commonly found in the gut of humans [29], can cause significant foodborne diseases [30]. Cholecystitis, septicemia, chronic obliterative, cholangitis, cystitis, traveler’s gastroenteritis, as well as other systemic diseases such as neonatal bacterial meningitis and pneumonitis, are the most frequent bacterial infections caused by *E. coli* [31]. Fatty acid synthesis is vital for bacterial cell viability and specificity [32]. Types I and II are the two major fatty acid synthesis types, with type I found in higher organisms such as mammals [33] and type II found in bacteria and plants. β-ketoacyl acyl carrier protein synthase (KAS) enzymes such as KAS I (FabB), KAS II (FabF), and KAS III (FabH) regulate the fatty acid synthesis initiation and elongation phases [34]. KAS I, which contains two identical homodimer subunits [33], belongs to the reducing enzyme group. KAS I is a His-His-Cys enzyme involved in fatty acid biosynthesis in bacteria and is responsible for enzyme-antibiotic interactions [32]. It can be a good therapeutic approach for designing new bioactive components because it is associated with the elongation of polyunsaturated fatty acids [31].

This study investigates the molecular interaction of fenchone with the selected targets mentioned above, which are important and related to cell membrane and cell wall synthesis, using in silico docking analysis. Further, the antibacterial, antifungal, and antibiofilm activity of fenchone was evaluated against *E. coli*, *P. aeruginosa,* and *C. albicans* using microbiological methods.

## 2. Results and Discussion

The current research mainly focuses on predicting the physicochemical parameters, molecular modeling, and docking analysis of fenchone molecules against three different proteins involved in antimicrobial infections.

### 2.1. Pharmacokinetic/ADME Properties of Fenchone

Several physicochemical traits and TPSA (Topological Polar Surface Area), a basic physiochemical parameter used to evaluate drug delivery characteristics, along with the pharmacokinetic property of fenchone, are predicted using Swiss ADME and are listed in Table 1.

The compounds showed high gastrointestinal (GI) absorption and are P-gp (p-glycoprotein) non-inhibitors that can also penetrate the blood–brain barrier (BBB). Fenchone inhibits the Cytochrome P450 isomers that possess a low on skin permeability of −4.73 cm/s. Drug likeness property was examined, which provides the molecules with a powerful drug based on molecular mass, Log P, hydrogen bond acceptors, and donor’s ratio. The compound violated any rules and disclosed a bioavailability score of around 0.55 (Table 1).

### 2.2. Physicochemical Properties of 1, 3-β–D-Glucan Synthase and Anthranilate-CoA Ligase

The physicochemical parameters and primary structure of 1, 3-β–D-glucan synthase and the Anthranilate-CoA ligase were computed using ExPasy ProtParam tools (Table 2). The presence of more non-polar amino acid residues suggests that the proteins are more hydrophobic [35]. The theoretical Pi values of 1, 3-D-glucan synthase and Anthranilate-CoA ligase sequences are 9.74 and 5.81, respectively, indicating the acidic nature of the sequence, which is useful in developing recombinant protein purification buffers [36]. The extinction coefficient (EC) values of 57,090 (1, 3-β–D-glucan synthase) and 57,130 (Anthranilate-CoA ligase) determine the quantitative investigation of protein–protein and protein–ligand interactions [37]. The sequence’s instability index indicates the protein’s stability; a score of less than 40 indicates that the protein is stable, whereas a score greater than 40 indicates it is unstable [38]. In the case of 1, 3-β–D-glucan synthase, the predicted structure is stable with an instability index value of 37.99, whereas the Anthranilate-CoA ligase had a value of 41.80. The addition of aliphatic amino acids to the side chain of a protein increases its thermal stability. The aliphatic index value for anti-freeze protein ranges from 57.83 to 125.23 [35], which is almost similar in both the proteins, indicating the protein could be stable for huge temperature variations.

### 2.3. Modeling of 3D Structure of Protein

**Figure 1:**
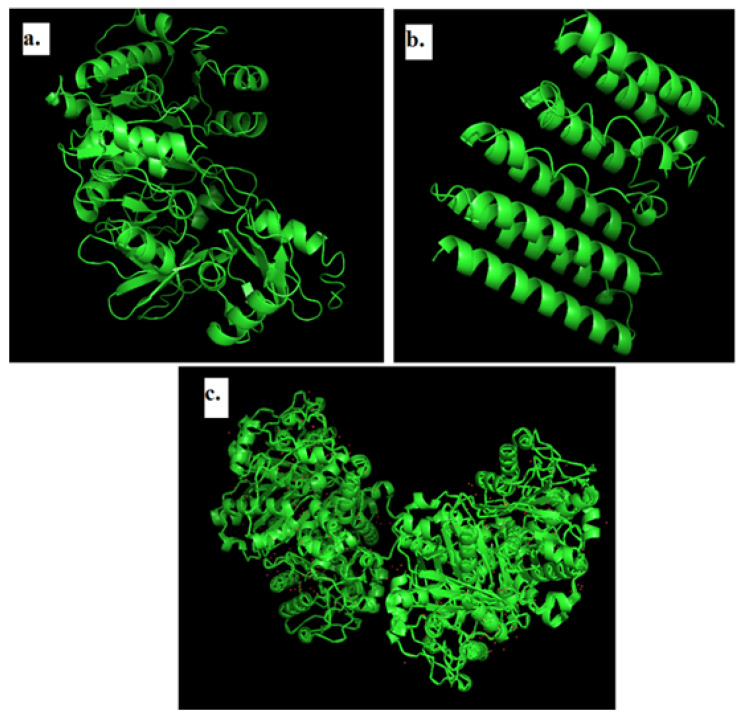
The 3D structure of modeled (**a**) Anthranilate-CoA ligase, (**b**) 1, 3-β–D-glucan synthase, and (**c**) β-ketoacyl acyl carrier protein synthase I (PDB:1FJ4).

Pymol was used to visualize the modeled 3D structure using I-TASSER and protein structures retrieved from the PDB (Figure 1). The I-Tasser theoretical tool that integrates ab initio modeling, threading, and atomic level structure modification technologies [39] is used to predict the three-dimensional protein structure of 1, 3-β–D-glucan synthase and the Anthranilate-CoA ligase.

I-Tasser was used to generate five models, each with a C-Score of −2.50, −3.01, −4.19, −5, and −5.00, and TM-Score and RMSD values of 0.42 ± 0.14 and 11.5 ± 4.5 Å for 1, 3-D-glucan synthase. In the case of the Anthranilate-CoA ligase, the C-Scores were 0.35, 0.07 and −0.49, whereas the TM-Score and RMSD values were 0.76 ± 0.10 and 6.6 ± 4.0 Å. These values are generated by the modeled structure’s threading pattern alignment and resolution parameter. The higher the C-Score, the more certain model 1 is, and it was selected for further study [40].

### 2.4. Structure Validation

**Figure 2:**
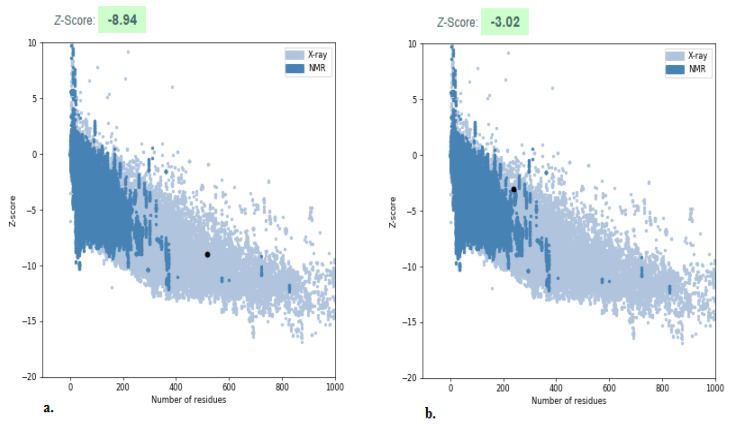
Identification of protein structure of Anthranilate-CoA ligase (**a**) and 1, 3-β–D-glucan synthase (**b**) using ProSA.

The predicted structure of the Anthranilate-CoA ligase and 1, 3-β–D-glucan synthase were validated by ProSA, which compares the Z-Score of the predicted model against structures refined from empirical techniques including X-ray and NMR (Figure 2). The total energy sandwiched in between the initial fold and misfolds determines the Z-Score of a protein in the ProSA web tool. The Z-Score primarily calculates the variation from the energy distribution produced by random conformation and determines the reliability of the modeled protein structure [41]. The Z-Score of the Anthranilate-CoA ligase is −8.94 and 1, 3-β–D-glucan synthase is −3.02, which had no significant variation from native structures of a similar size obtained from X-ray and NMR. Based on the ϕ and ψ angles between C_∞_–C and N–Cα, the position of amino acid residue in a segment is graphed on the Ramachandran plot [42]. The Ramachandran plot was employed in this study to investigate the predicted protein structure utilizing the SAVES Server. The statistical analysis of the modeling revealed that the majority of the residues fall in the most favored and additionally allowed region, including non-glycine and non-proline residues, which ensures good stereochemistry quality of the modeled structure [43].

**Figure 3:**
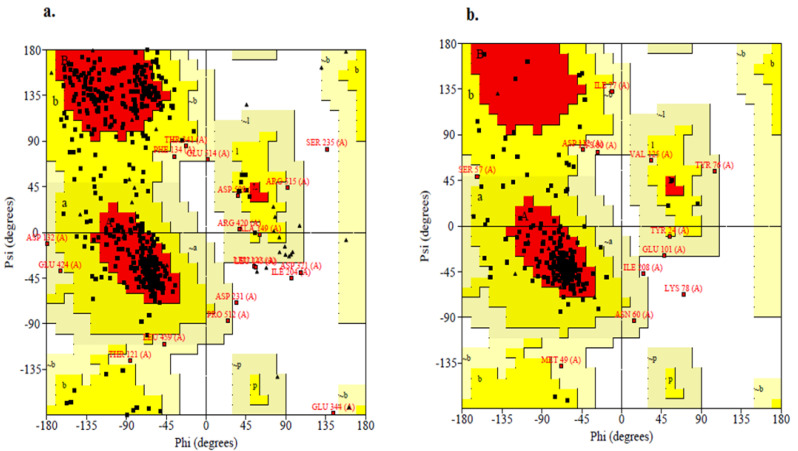
Assessing the quality of predicted Anthranilate-CoA ligase (**a**), and 1, 3-β–D-glucan synthase (**b**) structure.

Based on the position of amino acids present in the Anthranilate-CoA ligase, the geometry of the structure is shown in Figure 3a, which has 77.8% residue in the most superior sections, 18.9% in further acceptable sections, 2.5% in freely allowed sections, and 1.6% in prohibited sections. Similarly, the predicted 1, 3-β–D-glucan synthase had 76.7% residues in the most superior sections, 17.9% in further acceptable sections, 4.5% in freely allowed sections, and 0.9% in prohibited sections, which shows the geometry of the structure (Figure 3b).

### 2.5. Molecular Docking

**Figure 4:**
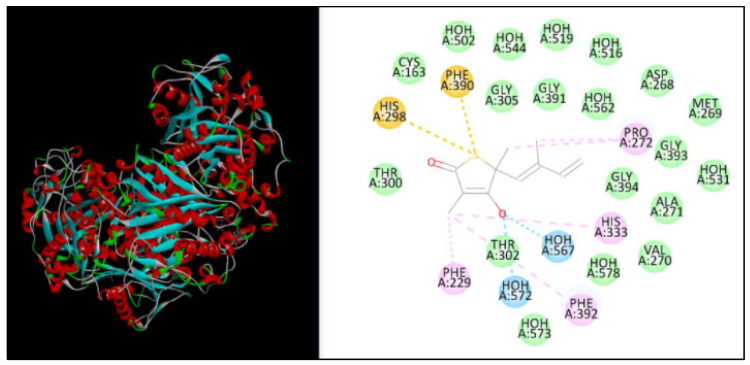
Interaction of β-ketoacyl acyl carrier protein synthase I with fenchone.

**Figure 5:**
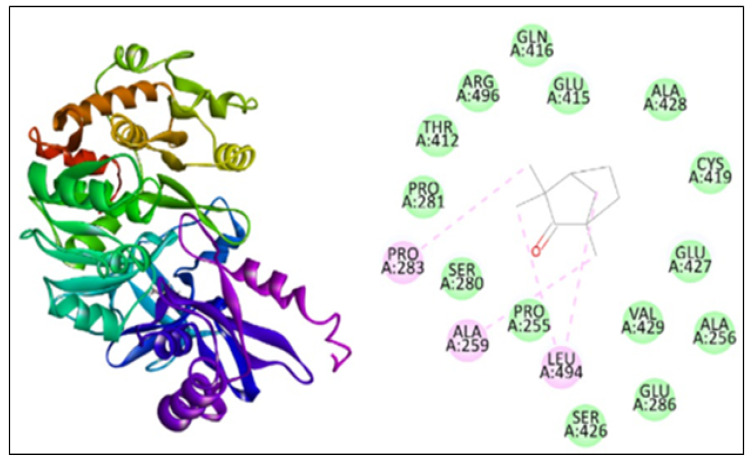
Interaction of Anthranilate-CoA ligase with fenchone.

**Figure 6:**
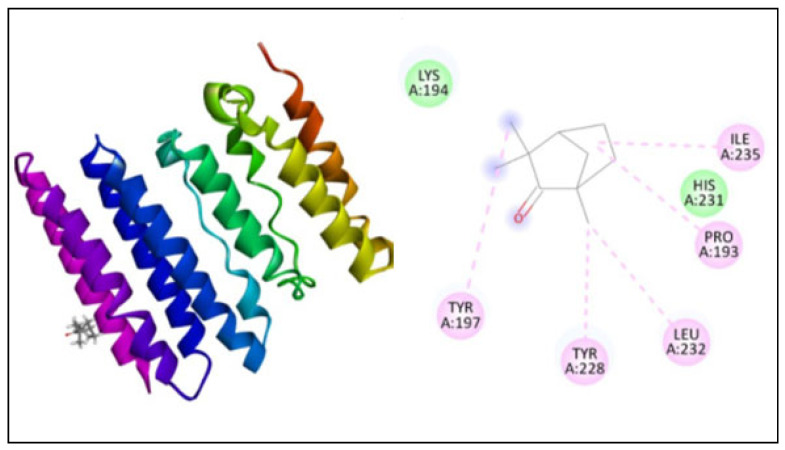
Interaction of 1, 3-β–D-glucan synthase with fenchone.

The docking study was performed using PatchDock for three different proteins, namely β-ketoacyl acyl carrier protein synthase I, the Anthranilate-CoA ligase, and 1, 3-β–D-glucan synthase with fenchone. The docking score, atomic content energy, hydrogen bonds, and amino acid residues involved in binding are listed in Table 3 along with the interactions in Figure 4, Figure 5 and Figure 6. The obtained results showed that the compound fenchone exhibited low ACE against the three used proteins. The possible reason for this could be the presence of functional side chains in the fenchone structure. The docking results of the molecules with the protein β-ketoacyl acyl carrier protein synthase I showed interactions with essential amino acids in the binding pocket with Pro272, as well as Van der Waals interactions with Phe390, Phe392, Phe229, Gly391, and, finally, a Pi-cation interaction with His298. The compound showed better interaction with the amino acid residues PRO283, ALA259, and LEU494, forming four hydrogen bond interactions with the Anthranilate-CoA ligase protein with an ACE value of −113.15 kcal/mol.

FKS1 expression is cell-cycle controlled and associated with cell wall reorganization, and catalytic subunits of glucan synthase (GS) are upregulated in cell wall construction [15]. Because FKS transcription factors are linked with the catalytic domain of GS, interrupting FKS1 lowers glucan synthase activity [44]. FKS and RHO1 proteins are known to be the preserved among fungi and are essential for cell survival [45]. As a result, inhibiting 1,3-β–D-glucan synthase disrupts cell wall construction and inhibits fungal growth. The docking results showed five interactions with TYR197, TYR228, LEU232, PRO193, and ILE235 with ACE −130.89 (kcal/mol).

### 2.6. Antimicrobial Activity of Fenchone

**Figure 7:**
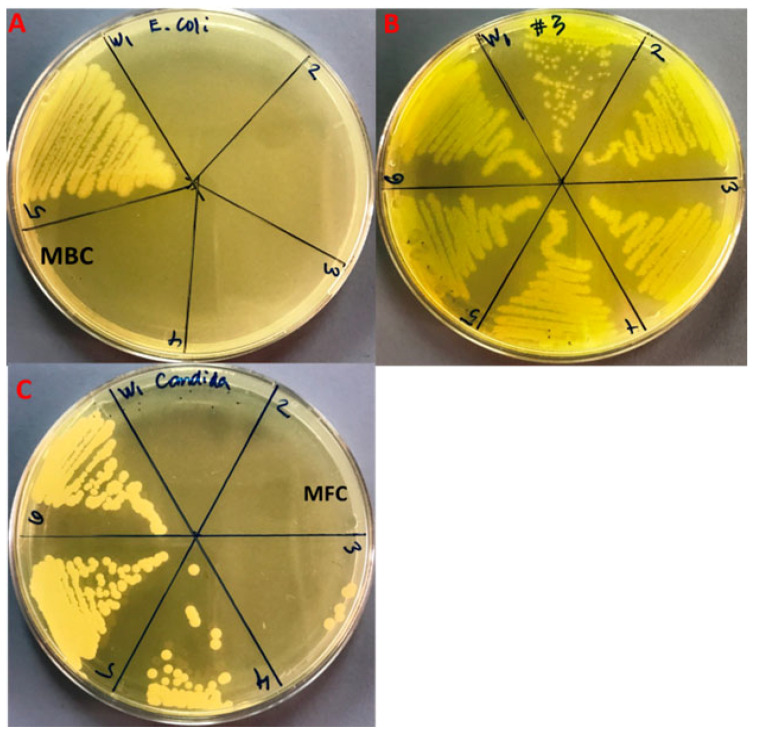
MBC/MFC (mg/mL) results of fenchone oil against *E. coli* (**A**), MDR-PA (**B**) and *C. albicans* (**C**). The dosage of fenchone oil is numerically depicted as 1, 2, 3, 4, 5 and 6, which are 200, 100, 50, 25, 12.5 and 6.25 mg/mL, respectively.

The rising development of antibiotic resistance, which leads to antimicrobial treatment insufficiency, is the fundamental challenge in antimicrobial chemotherapy. Overuse of antibiotics and the resulting antibiotic selection pressure are thought to be the most critical attributes significant to the emergence of different forms of antibiotic resistant bacteria [46], therefore, there is an immediate requirement to find new antimicrobial agents with various chemical structures and unique modes of action. Essential oils and their active components are broadly utilized in medicine as ingredients in a variety of medical products, as flavoring additives in food, and as perfumes in cosmetics [46]. The antimicrobial potential of fennel essential oil has been investigated against a variety of bacterial and fungal strains [46]. In the present study, the antibacterial and antifungal efficacy of commercial fenchone (a constituent of absinthe and the essential oil of fennel) against *E. coli*, MDR-PA, and *C. albicans* has been investigated by using the microbroth dilution method. The MIC/MBC/MFC values of fenchone against *E. coli,* MDR-PA, and *C. albicans* were shown in Table 4. The MIC and MBC results for Gram-negative bacterial, i.e., *E. coli*, and MDR-PA were found to be 8.3 ± 3.6/25 ± 0.0, and 266.6 ± 115.4/533.3 ± 230.9 mg/mL, respectively (Table 4; Figure 7A,B). Compared to Gram-negative MDR-PA, the MIC/MFC values of fenchone for *C. albicans* were found to be significantly lower, i.e., 41.6 ± 14.4/83.3 ± 28.7 mg/mL (Figure 7C). It was observed that fenchone has a significant effect on antimicrobial activity (*p* < 0.05). In a previous study, the lowest MIC values of fennel essential oil have been reported for *E. coli* and *C. albicans* [46]. Bassyouni et al. (2019) reported an MIC value of 0.78 to 6.25% against clinical isolates of C. albicans [47]. Kawther (2007) reported that the essential oil from fennel seeds exhibits prominent anticandidal activity against different *Candida* species [48]. It was reported that fennel essential oil and seed extracts have varying degrees of antimicrobial potential depending on the doses used [47,49]. Fennel essential oil has a higher sensitivity to Gram-negative and Gram-positive bacteria due to the occurrence of volatile compounds such as polyphenols in greater quantities [50]. It also enhances plasma membrane fluidity, which leads to greater fluid loss from bacterial species and inhibits microbial respiration [51,52]. As a result, the post-diffusion effect of essential oils on bacterial and fungal growth and metabolism appears to be the most important antibacterial activity [53,54]. Without a doubt, the essential oil of fenchone includes a plethora of interesting compounds that can be employed for medicinal purposes, primarily pharmacological potential [55]. Natural essential oils, similar to other plant products, provide broad-spectrum antibacterial action against pathogenic microbial strains. The present findings suggested that fenchone could be used as a natural antimicrobial treatment to treat various infections caused by pathogenic microorganisms and may provide pharmaceutical ingredients for the development of novel therapeutic and antimicrobial medicines.

### 2.7. Inhibition of Biofilm Formation

**Figure 8:**
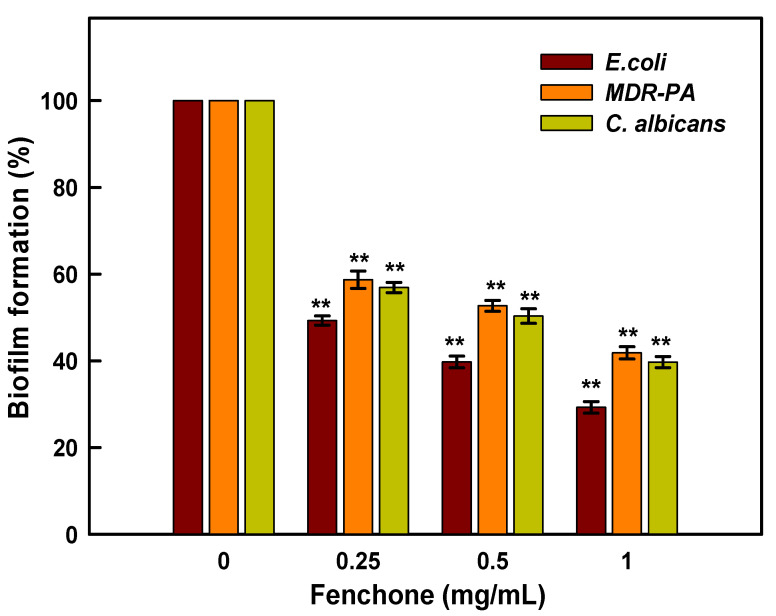
Impaired biofilm formation of *E. coli*, MDR-PA, and *C. albicans* caused by fenchone treatment. The data is presented as average of three independent tests in triplicates with standard deviation. The asterisks (**) represent the significance as *p* < 0.001 against their control experiments by One-Way ANOVA-based Pairwise Multiple Comparison Procedures (Holm-Sidak method), whereas overall significance level = 0.05 was conductedon SigmaPlot 11.05 statistical analysis software.

Biofilm production by *Candida* and bacterial spp is one of the most critical elements in wound infections, and it can lead to biofilm-related sepsis, which is the leading cause of wound-related death. The rate of antibiotic resistance is increasing due to the formation of organized bacterial and candida biofilm communities, which complicates the treatment therapy and leads to the development of chronic infection [56]. In recent decades, there has been a growing interest in exploring the diverse biological potential of plant metabolites. In this regard, plant-based essential oils have been suggested as a potential substitute for most commonly used antibiotics as well as an addition to conventional therapy. Essential oils derived from plants have been broadly used as antimicrobial, antioxidants, and flavoring agents due to the presence of various secondary metabolites such as terpenoids, alkaloids, and polyphenolic compounds [57]. The antibacterial, antifungal, and antibiofilm properties of a large number essential oils have been demonstrated against a diverse variety of pathogenic bacteria [57]. Despite numerous studies evaluating a variety of biological activities, none have looked at fenchone’s antibiofilm potential against bacteria and Candida biofilms. Therefore, the aim of this study was to assess the potency of fenchone against *E. coli*, MDR-PA, and *C. albicans*. The result in Figure 8 demonstrates the inhibition of biofilm production of *E. coli*, MDR-PA, and *C. albicans* by fenchone at varying concentrations. It has been observed that *E. coli*, MDR-PA, and *C. albicans* biofilms treated with a lower concentration of fenchone, i.e., 0.25 mg/mL, inhibit biofilm formation by 51.7, 40.85, and 44.27%, respectively. Whereas, under similar conditions, fenchone at 1.0 mg/mL inhibits biofilm production of *E. coli*, MDR-PA, and *C. albicans* by 71.03, 59.15, and 61.71%, respectively. The data is presented as the average of three independent tests in triplicates with standard deviation. The asterisks (**) represent the significance as *p* < 0.001 against their control experiments by One-Way ANOVA-based Pairwise Multiple Comparison Procedures (Holm-Sidak method), whereas the overall significance level = 0.05, was conducted on SigmaPlot 11.05 statistical analysis software (Figure 8). Previously, it has been reported that fennel essential oil significantly decreased the formation of *S. aureus* biofilm and reduced the metabolic activity of attached cells as well [56]. In another study, it has been investigated that fennel oil at concentrations ranging from 6.25 to 25% reduces the biofilm formation in *C. albicans* by 50% [47]. Whereas, in the present study, it has been found that fenchone inhibits biofilm formation in *C. albicans* by 61.71%, which is much lower than the previous report [47].

## 3. Materials and Methods

### 3.1. Retrieval of the Ligand Molecule and Protein Structure for ADME Studies

The 3D structure of the fenchone compound was retrieved from the Pub Chem database using the Swiss ADME web server (Swiss Institute of Bioinformatics, Lausanne, Switzerland) [58], and the Simplified Molecular Input Line Entry System (SMILES) format of the compounds was used to develop their ADME/pharmacokinetic profile and drug-likeness characteristics. The Protein Data Bank (PDB) is a collection of 3-D structural data for essential biological substances [59]. A 3D structure for β-ketoacyl acyl carrier protein synthase I (KAS I), which is involved in cell wall formation, was discovered using the Protein Data Bank.

### 3.2. Molecular Docking Analysis

The sequences of 1, 3-β–D-glucan synthase and Anthranilate-CoA ligase protein were retrieved from the Swiss-Prot database. The ProtParam application was used to weigh the physicochemical characteristics of the obtained protein sequence by using the Expasy web browser. The 3D structures of the protein sequences were developed using the I-Tasser server. Based on the C-Score (−5 to +2), TM-score, and RMDS, the accuracy of the generated protein structure was estimated. The quality of the model was validated using the structural validation algorithm ProSA, and the overall structure geometry was calculated using the Ramachandran plot and the SAVES Server (https://saves.mbi.ucla.edu/; accessed on 11 November 2021).

Molecular modeling investigation was conducted for the retrieved 3D structure and modeled protein structure using the PatchDock online server and the interactions are visualized using Discovery Studio.

### 3.3. Evaluation of Antibacterial and Antifungal Activity

The antimicrobial activity of fenchone was investigated against *E. coli*, *C. albicans,* and multidrug resistant *Pseudomonas aeruginosa* (MDR-PA). Fenchone [(+)-fenchone] was obtained from Alfa Aesar with 98% purity. Bacterial cultures were grown for 24 h in a shaker incubator in Luria Bertani (LB) broth at 37 °C, whereas *C. albicans* was grown in Sabouraud Dextrose Broth (SD) broth at 28 °C. In the following step, the bacterial culture was rinsed with phosphate buffer saline, and the pellet was collected and re-suspended in fresh LB broth.

### 3.4. Minimal Inhibitory Concentration (MIC)

The minimum inhibitory concentration (MIC) value of fenchone was investigated by the standard microbroth dilution method in a 96-well microtiter plate. Briefly, 20 µL of freshly grown cultures of each tested organism was inoculated in 180 µL of Brain Heart Infusion (BHI) broth containing a two-fold concentration (800–1.56 mg/mL) of fenchone for 24 h at 37 °C. MICs are defined as the low concentration of a tested compound at which no obvious growth was observed [60]. Penicillin and fluconazole were used as reference drugs, and it was found that multidrug resistant *P. aeruginosa* was resistant to penicillin. For *E. coli*, the MIC value for Penicillin was 64 ug/mL, whereas fluconazole showed a 64 ug/mL MIC for *C. albicans*.

### 3.5. Minimal Bacterial and Fungicidal Concentration (MBC and MFC)

To determine the MBCs and MFCs, a 10µL suspension of bacteria and *Candida* was taken from microtiter plate wells that had no bacterial and fungal growth and spread on Mueller Hinton Agar (MHA) and Sabouraud Dextrose Agar (SDA) plates, respectively, for 24 h. MBCs/MFCs values of the tested compound could be defined as the minimum concentration of tested fenchone at which no bacterial or fungal growth or less than 3 CFUs were detected [60].

### 3.6. Evaluation of Anti-Biofilm Activity

Polystyrene flat bottom microtiter tissue culture was used to determine the anti-biofilm efficacy of fenchone against *E. coli*, MDR-PA, and *C. albicans* biofilms [61]. Briefly, 20 µL of fresh culture was added to 180 µL of LB broth containing different concentrations of fenchone and incubated for 24 h at 37 °C. After that, the content of the plate was discarded and washed three times with PBS before being stained with crystal violet dye (0.1% *w*/*v*) for 15 min. The excess dye was washed off and followed by rinsing in PBS and drying. The ethanol (95%) was then used to solubilize the stain, and the biofilm production was then measured at OD595 using an ELISA reader.

### 3.7. Statistical Analysis

All the obtained results were analyzed statistically using SPSS software version 20.0 (SPSS Inc., Chicago, IL, USA) and Microsoft Excel 2010 (Microsoft Corporation, Redmond, WA, USA).

## 4. Conclusions

In silico analysis revealed that the peptide-ketoacyl acyl carrier protein synthase I from *E. coli* and the anthranilate-CoA ligase from *P. aeruginosa* have favorable stereochemical, physiochemical, and functional properties. The fenchone molecule showed four H-bond interactions with the amino acid residues PRO283, ALA259, and LEU494 of anthranilate-CoA ligase protein with an ACE value of −113.15 kcal/mol. The docking results of fenchone with 1, 3-β–D-glucan synthase of *C. albicans* had five H-bond interactions with amino acid residues TYR197, TYR228, LEU232, PRO193, and ILE235 that have an ACE value of −130.89 (kcal/mol). The preliminary antibacterial, antifungal, and antibiofilm screening results demonstrated that the antimicrobial and antibiofilm potency of fenchone molecules was found to be moderate to high and were effective against *E. coli, P. aeruginosa,* and *C. albicans*. Among the tested pathogens, *E. coli* was the most sensitive to fenchone, showing the lowest MIC/MBC (i.e., 6.25 and 25 mg/mL). In addition, it has also been observed that fenchone had a significant effect on the biofilm forming abilities of tested pathogens. These findings suggested that the fenchone molecule has biological significance and might be explored as a new antimicrobial agent. However, additional electron microscopic and molecular research is still required to completely understand the mode of action of fenchone against bacteria and candida in order to justify the real-world applications of fenchone as a natural antimicrobial agent.

## Figures and Tables

**Table 1:** Pharmacokinetic properties and physicochemical characteristics of Fenchone.

S.No.	Pharmacokinetic/ADME Properties	Fenchone
1	GI absorption	High
2	BBB permeant	Yes
3	P-gp substrate	No
4	CYP1A2 inhibitor	No
5	CYP2C19 inhibitor	No
6	CYP2C9 inhibitor	No
7	CYP2D6 inhibitor	No
8	CYP3A4 inhibitor	No
9	Log Kp (skin permeation)	−4.73 cm/s
10	Lipinski violations	0
11	Bioavailability Score	0.55
12	Number of rotatable bonds	0
13	Num. H-bond acceptors	1
14	Num. H-bond donors	0
15	Molar Refractivity	45.64
16	Topological Polar Surface Area	17.07 Å^2^

**Table 2:** Physicochemical parameters of 1, 3-β–D-glucan synthase and Anthranilate-CoA ligase investigated in this study.

ProtParam Parameters	Values	
	1, 3-β–D-Glucan Synthase	Anthranilate-CoA Ligase
Amino acids	240	517
Molecular weight	28,162.98	56,607.52
Theoretical Pi	9.74	5.81
Amino acidcomposition (%)	Ala =5.0%, Arg = 5.0%, Asn = 3.3%, Asp = 2.5%, Cys = 1.7%, Gln = 2.1%, Glu = 2.1% Gly = 3.3%, His = 0.8%, Ile = 10.4%, Leu = 14.2%, Lys = 5.8% Met = 4.2%, Phe = 7.1%, Pro =2.9%, Ser = 6.7%, Thr = 5.8% Trp = 2.5%, Tyr = 6.7%, Val = 7.9%	Ala = 11.8%, Arg = 8.1%, Asn = 2.3%, Asp = 6.0%, Cys = 2.3% Gln = 4.1%, Glu = 5.2%, Gly = 7.5%, His = 2.7%, Ile = 2.7%, Leu = 14.1%, Lys = 1.2%, Met = 1.0%, Phe = 4.4%, Pro =7.0%, Ser = 6.2%, Thr = 4.1%, Trp =1.4%, Tyr = 2.3%, Val = 5.6%
Negatively charged residues	11	58
Positively charged residues	26	48
Atomic composition	Carbon 1339 Hydrogen 2079 Nitrogen 313 Oxygen 322 Sulfur 14	Carbon 2525 Hydrogen 3949 Nitrogen 717 Oxygen 732 Sulfur 17
Formula	C_1339_H_2079_N_313_O_322_S_14_	C_2525_H_3949_N_717_O_732_S_17_
Total number of atoms	4067	7940
Extinction coefficient	57,090 Abs 0.1% (=1 g/L) 2.027, assuming all pairs of Cys residues form cysteine	57,130 Abs 0.1% (=1 g/L) 1.009, assuming all pairs of Cys residues form cysteine
Calculated half-life	5.5 h (in vitro—mammalian reticulocytes). 3 min (in vivo—yeast). 2 min (in vivo—*E. coli).*	0 h (in vitro—mammalian reticulocytes). >20 h (in vivo—yeast). >10 h (in vivo—*E. coli*).
Instability index	37.99 (This indicates that the peptide is stable).	41.80 (This indicates that the peptide is unstable).
Aliphatic index	123.83	93.69
Grand average of hydropathicity (GRAVY)	0.656	−0.068

**Table 3:** The docking interaction parameters of Fenchone with three proteins.

Receptor	PatchDock Score	Atomic Content Energy (ACE) (kcal/mol)	No. of H-Bonds	Amino Acid Residues
β-ketoacyl acyl carrier protein synthase I	2794	−131.30	7	HIS298, PHE390, PRO272, HIS333, PHE229, PHE392
Anthranilate-CoA ligase	3412	−113.15	4	PRO283, ALA259, LEU494
1, 3-β–D-glucan synthase	3124	−130.89	5	TYR197, TYR228, LEU232, PRO193, ILE235

**Table 4:** MIC and MBC (mg/mL) values of Fenchone against tested pathogen.

Strains	MIC	MBC
*E. coli*	8.3 ± 3.6 ^a^	25.0 ± 0.0 ^d^
MDR-*P. aeruginosa*	266.6 ± 115.4 ^b^	533.3 ± 230.9 ^e^
*C. albicans*	41.6 ± 14.4 ^c^	83.3 ± 28.7 ^f^

Note: The statistically significant difference between MICs ^a^ and ^b^, ^b^ and ^c^, and MBCs ^d^ and ^e^ and, ^e^ and ^f^, were found to be as *p* < 0.010 and *p* < 0.013, and *p* < 0.011 and *p* < 0.013, respectively, whereas, overall significance level = 0.05.

## Data Availability

The authors confirm that the data supporting the study’s findings are included in the article.

## References

[1] Aslam B., Wang W., Arshad M.I., Khurshid M., Muzammil S., Rasool M.H., Nisar M.A., Alvi R.F., Aslam M.A., Qamar M.U. (2018). Antibiotic resistance: A rundown of a global crisis. Infect. Drug Resist..

[2] Badgujar S.B., Pate V.V., Bandivdekar A.H. (2014). *Foeniculum vulgare* Mill: A Review of Its Botany, Phytochemistry, Pharmacology, Contemporary Application, and Toxicology. BioMed. Res. Int..

[3] Choi E.M., Hwang J.K. (2004). Anti-Inflammatory, Analgesic and Antioxidant Activities of The Fruit of *Foeniculum vulgare*. Fitoterapia.

[4] Keskin I., Gunal Y., Ayla S., Kolbasi B., Sakul A., Kilic U., Gok O., Koroglu K., Ozbek H. (2017). Effects of *Foeniculum vulgare* essential oil compounds, fenchone and limonene, on experimental wound healing. Biotech. Histochem..

[5] Ozbek H. (2007). Investigation of lethal dose levels and anti-inflammatory effect of Fenchone. Turk. Hij ve Deney Biyol Dergisi..

[6] Rohman F., Putra W.E. (2021). The bioinformatics perspective of *Foeniculum vulgare* fruit’s bioactive compounds as natural anti-hyperglycemic against alpha-glucosidase. Biodivers. J..

[7] Singh S., Gupta P., Gupta J. (2020). Virtual Structural Similarity Elucidates Bioactivity of Fenchone: A Phytochemical Enriched in Fennel Essential Oil. Curr. Drug Discov. Technol..

[8] Him A., Ozbek H., Turel I., Oner A.C. (2008). Antinociceptive activity of alpha-pinene and fenchone. Pharmacologyonline.

[9] Pappas P.G., Kauffman C.A., Andes D.R., Clancy C.J., Marr K.A., Ostrosky-Zeichner L., Reboli A.C., Schuster M.G., Vazquez J.A., Walsh T.J. (2016). Clinical practice guideline for the management of candidiasis: 2016 update by the infectious diseases society of America. Clin. Infect. Dis..

[10] Wächtler B., Citiulo F., Jablonowski N., Förster S., Dalle F., Schaller M., Wilson D., Hube B. (2012). *Candida albicans*-epithelial interactions: Dissecting the roles of active penetration, induced endocytosis and host factors on the infection process. PLoS ONE.

[11] Pfuller R., Graser Y., Erhard M., Groenewald M. (2011). A novel flucytosineresistant yeast species, *Candida pseudoaaseri*, causes disease in a cancer patient. J. Clin. Microbiol..

[12] Brown G.D., Gordon S. (2005). Immune recognition of fungal beta-glucans. Cell Microbiol..

[13] Houšť J., Spížek J., Havlíček V. (2020). Antifungal Drugs. Metabolites.

[14] Andriole V.T., Bodey G.P., Springfield N.J. (1994). Pocket Guide to Systemic Antifungal Therapy.

[15] Denning D.W. (2003). Echinocandin antifungal drugs. Lancet.

[16] Hector R.F. (1993). Compounds active against cell walls of medically important fungi. Clin. Microbiol Rev..

[17] Bowman S.M., Free S.J. (2006). The structure and synthesis of the fungal cell wall. BioEssays.

[18] Page M.G., Heim J. (2009). Prospects for the next anti-Pseudomonas drug. Curr. Opin. Pharmacol..

[19] Zavascki A.P., Carvalhaes C.G., Picao R.C., Gales A.C. (2010). Multidrugresistant *Pseudomonas aeruginosa* and *Acinetobacter baumannii: Resistance* mechanisms and implications for therapy. Exp. Rev. Anti Infect. Ther..

[20] Strateva T., Yordanov D. (2009). *Pseudomonas aeruginosa*—A phenomenon of bacteria resistance. J. Med. Microbiol..

[21] Moradali M.F., Ghods S., Rehm B.H. (2017). Pseudomonas aeruginosa lifestyle: A paradigm for adaptation, survival, and persistence. Front. Cell. Infect. Microbiol..

[22] De Kievit T.R. (2009). Quorum sensing in *Pseudomonas aeruginosa* biofilms. Environ. Microbiol..

[23] Thi M.T., Wibowo D., Rehm B.H. (2020). *Pseudomonas aeruginosa* biofilms. Int. J. Mol. Sci..

[24] Castillo-Juárez I., Maeda T., Mandujano-Tinoco E.A., Tomás M., Pérez-Eretza B., García-Contreras S.J., Wood T.K., García-Contreras R. (2015). Role of quorum sensing in bacterial infections. World J. Clin. Cases.

[25] Waters C.M., Bassler B.L. (2005). Quorum sensing: Cell-to-cell communication in bacteria. Annu. Rev. Cell Dev. Biol..

[26] Xiao G., Déziel E., He J., Lépine F., Lesic B., Castonguay M.H., Milot S., Tampakaki A.P., Stachel S.E., Rahme L.G. (2006). MvfR, a key *Pseudomonas aeruginosa* pathogenicity LTTR-class regulatory protein, has dual ligands. Mol. Microbiol..

[27] Grandclément C., Tannières M., Moréra S., Dessaux Y., Faure D. (2016). Quorum quenching: Role in nature and applied developments. FEMS Microbiol Rev..

[28] Shaker B., Ahmad S., Thai T.D., Eyun S.I., Na D. (2020). Rational Drug Design for *Pseudomonas aeruginosa* PqsA Enzyme: An in silico Guided Study to Block Biofilm Formation. Front. Mol. Biosci..

[29] Singleton P. (1999). Bacteria in biology. Biotechnology and Medicine.

[30] Vogt R.L., Dippold L. (2005). *Escherichia coli* O157:H7 outbreak associated with consumption of ground beef. Public Health Rep..

[31] Sabbagh G., Berakdar N. (2015). Docking studies of flavonoid compounds as inhibitors of β-ketoacyl acyl carrier protein synthase I (Kas I) of *Escherichia coli*. J. Mol. Graph. Model..

[32] Price A.C., Choi K.H., Heath R.J., Li Z., White S.W., Rock C.O. (2001). Inhibition of β-ketoacyl-acyl carrier protein synthases by thiolactomycin and cerulenin: Structure and mechanism. J. Biol. Chem..

[33] Richard J.H., Suzane J., Charles O.R. (2002). Fatty Acid and Phospholipid Metabolismin Prokaryotes. Biochemisty of Lipids, Lipoproteins and Membranes.

[34] Zhang H., Machutta C.A., Tonge P.J. (2010). Fatty Acid Biosynthesis and Oxidation.

[35] Sivakumar K., Balaji S., Gangaradhakrishna N. (2007). In silico characterization of antifreeze proteins using computational tools and servers. J. Chem. Sci..

[36] Gasteiger E., Hoogland C., Gattiker A., Duvaud S., Wilkins M.R., Appel R.D., Bairoch A., John M.W. (2005). Protein Identification and Analysis Tools on the ExPASy Server. The Proteomics Protocols Handbook.

[37] Chhetri A., Loksztejn A., Yokoyama K. (2021). Quantitative Characterization of the Amount and Length of (1,3)-β-d-glucan for Functional and Mechanistic Analysis of Fungal (1,3)-β-d-glucan Synthase. Bio Protoc..

[38] Sivakumar K., Lazakidou A. (2010). Biocomputation and Biomedical Informatics: Case Studies and Applications.

[39] Zhou H., Skolnick J. (2007). Ab initio protein structure prediction using chunk-TASSER. Biophys. J..

[40] Yang J., Zhang Y. (2015). Protein Structure and Function Prediction Using I-TASSER. Curr. Protoc. Bioinform..

[41] Wiederstein M., Sippl M.J. (2007). ProSA-web: Interactive web service for the recognition of errors in three-dimensional structures of proteins. Nucleic Acids Res..

[42] Yadav M., Alka S., Sushma R., Eva L. (2010). Structural modeling and simulation studies of *Brugia malayi* glutathione-S-transferase with compounds exhibiting antifilarial activity: Implications in drug targeting and designing. J. Mol. Graph. Model..

[43] Hasan M.A., Mazumder H.H., Chowdhury A.S., Datta A., Khan M.A. (2015). Molecular-docking study of malaria drug target enzyme transketolase in Plasmodium falciparum 3D7 portends the novel approach to its treatment. Biol. Med..

[44] Douglas C.M., Foor F., Marrinan J.A., Morin N., Nielsen J.B., Dahl A.M., Baginsky W., Li W., EL-Sherbeini M., Clemas J.A. (1994). The Saccharomyces cerevisiae FKSI (ETGJ) gene encodes an integral membrane protein which is a subunit of 1,3-,8-Dglucan synthase. Proc. Natl. Acad. Sci. USA.

[45] Beauvais A., Bruneau J.M., Mol P.C., Buitrago M.J., Legrand R., Latge J.P. (2001). Glucan synthase complex of *Aspergillus fumigatus*. J. Bacteriol..

[46] Gulfraz M., Mehmood S., Minhas N., Jabeen N., Kausar R., Jabeen K., Arshad G. (2008). Composition and antimicrobial properties of essential oil of *Foeniculum vulgare*. Afr. J. Biotechnol..

[47] Bassyouni R.H., Wali I.E., Kamel Z., Kassim M.F. (2019). Fennel oil: A promising antifungal agent against biofilm forming fluconazole resistant *Candida albicans* causing vulvovaginal candidiasis. J. Herb. Med..

[48] Kawther F.A. (2007). Antimicrobial Activity of Essential Oils of some Medicinal Plants from Saudi Arabia. Saudi J. Biol Sci..

[49] Roby M.H., Sarhan M.A., Selim K.A., Khalel K.I. (2013). Antioxidant and antimicrobial activities of essential oil and extracts of fennel (*Foeniculum vulgare* L.) and chamomile (*Matricaria chamomilla* L.). Ind. Crops Prod..

[50] Medina E., Castro D., Romero A.C., Brenes M. (2006). Comparison of the Concentrations of Phenolic Compounds in Olive Oils and Other Plant Oils: Correlation with Antimicrobial Activity. J. Agric. Food Chem..

[51] Lamber R.J., Skandamis P.N., Coote P.J., Nycas G.J. (2001). A Study of the Minimum Inhibitory Concentration and Mode of Action of Oregano Essential Oil, Thymol and Carvacrol. J. Appl Microbiol..

[52] Popović V.B., Petrović S.D., Milenković M.T., Drobac M.M., Couladis M.A., Niketić M.S. (2015). Composition and Antimicrobial Activity of the Essential Oils of *Laserpitium latifolium* L. and *L. ochridanum* Micevski (Apiaceae). Chem. Biodivers..

[53] Walsh S.E., Maillard J.Y., Russell A.D., Catrenich C.E., Charbonneau D.L., Bartolo R.G. (2003). Activity and Mechanism of Action of Selected Biocidal Agents on Gram-Positive and -Negative Bacteria. J. Appl. Microbiol..

[54] Upadhyay R.K., Dwivedi P., Ahmad S. (2011). Antifungal Activity of 16 Plant Essential Oils against *S. cerevisiae*, *Rhizopus stolonifer* and *Aspergillus flavus*. J. Pharm. Res..

[55] Upadhyay R.K., Dwivedi P., Ahmad S. (2010). Screening of Antibacterial Activity of Six Plant Essential Oils against Pathogenic Bacterial Strains. Asian J. Med. Sci..

[56] Kwiatkowski P., Grygorcewicz B., Pruss A., Wojciuk B., Giedrys-Kalemba S., Dołêgowska B., Zielińska-Bliźniewska H., Olszewski J., Sienkiewicz M., Kochan E. (2020). Synergistic effect of fennel essential oil and hydrogen peroxide on bacterial biofilm. Postȩpy Dermatol. Alergol..

[57] Kim Y.G., Lee J.H., Gwon G., Kim S.I., Park J.G., Lee J. (2016). Essential oils and eugenols inhibit biofilm formation and the virulence of *Escherichia coli* O157: H7. Sci. Rep..

[58] Daina A., Zoete V. (2017). Swiss ADME: A free web tool to evaluate pharmacokinetics, druglikeness and medicinal chemistry friendliness of small molecules. Sci. Rep..

[59] Berman H.M. (2008). The Protein Databank: A historical perspective. Acta Cryst..

[60] Ansari M.A., Kalam A., Al-Sehemi A.G., Alomary M.N., AlYahya S., Aziz M.K., Srivastava S., Alghamdi S., Akhtar S., Almalki H.D. (2021). Counteraction of Biofilm Formation and Antimicrobial Potential of Terminalia catappa Functionalized Silver Nanoparticles against Candida albicans and Multidrug-Resistant Gram-Negative and Gram-Positive Bacteria. Antibiotics.

[61] Ansari M.A., Akhtar S., Rauf M.A., Alomary M.N., AlYahya S., Alghamdi S., Almessiere M.A., Baykal A., Khan F., Adil S.F. (2021). Sol–Gel Synthesis of Dy-Substituted Ni0. 4Cu0. 2Zn0. 4 (Fe2-xDyx) O4 Nano Spinel Ferrites and Evaluation of Their Antibacterial, Antifungal, Antibiofilm and Anticancer Potentialities for Biomedical Application. Int. J. Nanomed..

